# Drama-based simulations in first aid education: psychometric validation of a student assessment tool in school settings

**DOI:** 10.3389/fpubh.2026.1710189

**Published:** 2026-03-02

**Authors:** Cristina Dumitru, Konstantinos Mastrothanasis, Emmanouil Pikoulis, Maria Kladaki, Konstantinos Zervoudakis, Celal Deha Dogan, Ipek Gonullu, Maria Magdalena Stan, Evika Karamagioli, Maria Ledaki, Panagiotis Alexopoulos, Despoina Papantoniou, Anastasia Pikouli, Sotirios Papantoniou, Sengul Erden

**Affiliations:** 1 Department of Education, The National University of Science and Technology Politehnica Bucharest, Pitești University Centre, Pitesti, Arges, Romania; 2 School of Medicine, National and Kapodistrian University of Athens, Goudi, Athens, Greece; 3 School of Health Sciences, Faculty of Medicine, National and Kapodistrian University of Athens (NKUA), Goudi, Athens, Greece; 4 Department of Primary Education, University of the Aegean, Rhodes, Greece; 5 School of Production Engineering and Management, Technical University of Crete, Chania, Greece; 6 Department of Measurement and Evaluation, Faculty of Educational Sciences, Ankara University, Ankara, Türkiye; 7 Department of Medical Education and Informatics, Faculty of Medicine, Ankara University, Ankara, Türkiye; 8 Department of Neurology, School of Medicine, National and Kapodistrian University of Athens, Athens, Greece; 9 Medical School, University of Nicosia, Nicosia, Cyprus

**Keywords:** drama-based pedagogy, emergency preparedness education, first aid education, psychometric validation, role-play, role-playing, simulated patients

## Abstract

**Introduction:**

Drama-based simulations and role-playing are increasingly integrated into school-based first aid education, as they enable students to rehearse emergency response skills within controlled and realistic environments. Despite their pedagogical value, the systematic assessment of simulated patient performance in school contexts remains underdeveloped, particularly for younger populations. This study aimed to adapt and psychometrically validate the Standardized Patient Performance Rating Scale - Student Version (SPRS-S) for use in first aid training with school-aged students in Greece.

**Methods:**

The study included 332 students aged 10-16 years (160 boys, 172 girls) and three first aid instructors. The scale underwent translation and back-translation procedures to ensure linguistic equivalence and contextual appropriateness within the Greek educational setting. Construct validity was examined through exploratory and confirmatory factor analyses, while internal consistency was assessed using reliability indices. In addition, an unsupervised computational intelligence clustering algorithm was implemented to classify simulated patient performance into distinct profiles.

**Results:**

Factor analyses confirmed a unidimensional structure with satisfactory reliability coefficients. The Greek version of the SPRS-S demonstrated clarity, internal coherence, and measurement stability across demographic subgroups. The clustering analysis identified three distinct performance profiles among simulated patients, providing additional interpretive depth regarding role enactment quality.

**Discussion:**

The validated Greek version of the SPRS-S constitutes a psychometrically robust instrument for evaluating simulated patient performance in school-based first aid education. Its application supports structured feedback processes and strengthens the pedagogical use of role-playing simulations. The findings support the systematic evaluation of drama-based health education and extend assessment practices to school-aged populations.

## Introduction

First aid training is a way to improve readiness and response in emergencies (1). Its importance is clear during disaster situations, where quick actions and structured management must be taken to protect communities. Receiving training in first aid does not concern professional rescuers alone but also members of the general population, who should also be able to provide assistance to others who are in need until help arrives (2). These skills are considered to be important not only during natural disasters but also in sudden daily crucial situations like accidents or medical emergencies.

In this context, learning to act quickly in emergencies is essential and it is not just for adults (3). For this reason, efforts are made, and schools have already started teaching it, to ensure that students can handle an emergency situation (4). In addition, this training also enhances social responsibility and teamwork, it helps people in managing crises, and improves community safety (5).

Modern teaching methods follow effective practices by emphasizing experiential, hands-on learning through activities like drama-based simulations and role-playing (6–13). These approaches allow students to practice in safe settings that mimic real situations without the risks that can occur during actual emergencies (3, 14–17). Simulations provide a safe environment while allowing participants to gain practical experience through hypothetical scenarios (7, 18). At the same time, they help in developing social and emotional skills needed for managing emergencies effectively (19, 20).

A highly effective method of simulation-based first aid training involves the use of standardized patients or simulated victims (21). Those simulated victims are trained individuals who recreate scenarios that require quick intervention, allowing trainees to practice first aid and communication in controlled settings, while at the same time they are getting feedback that helps them to refine their skills before encountering real emergencies.

In simulation, role-playing functions as an experiential learning method that allows trainees to engage in realistic scenarios without the risk of harm, supporting the achievement of educational goals (7, 10, 22–27). When trainees interact with simulated victims they get effective training and feedback in a controlled environment, which allows learners to be taught and apply their skills without experiencing the anxiety associated with real emergencies (28). Moreover, this particular training approach is based on the principles of situated learning (29), social constructivism (30), and game-based learning (31, 32), and it provides learners with meaningful experiential learning opportunities in the context of a demanding and challenging controlled environment.

Assessing and monitoring these simulations is very important for making sure that the quality of the educational process is effective (33). Especially in simulations of emergency scenarios, feedback from trainee interactions with the simulated victims helps identify possible training strengths or areas for improvement, which makes the evaluation process an important tool for successful training and learning (34). However, the existing scales in this field are designed solely to evaluate interactions between the simulated patients and the medical or nursing students (35) and most of the time they are time-consuming and less appropriate for the specific needs of students who participate in those programs. As a result, it is important for trainers to have a valid and reliable scale for first aid training in schools. This scale should be based on well-established standardized tools that have already been used in medical education to evaluate simulations and role-playing.

Since there are no validated measurement tools for first aid education in this population, there is a need for appropriate assessment instruments tailored to school-based first aid training. For this purpose, a modern and short scale, like the one by Gonullu et al. (34), was selected for the psychometric adaptation and standardization, to ensure a reliable evaluation tool personalized to the specific requirements of first aid education for students.

## Simulation-based first aid training through standardized patients and role-playing

First aid training helps people to respond to emergencies, including natural disasters, medical issues, and everyday accidents. An important element of this training is the use of simulated patients or victims (21). These people are trained to represent different physical or psychological conditions that need fast response (36). Moreover, their purpose is to provide a safe learning environment that feels real, where trainees can practice first aid skills and get helpful feedback (37).

This teaching method, which involves students through standardized patients, simulations, and role-playing in an active way, is considered to be effective and is supported by several learning theories (28, 38). The main theoretical frameworks include Situated Learning (29), Social Constructivism (30), and Game-Based Learning (31).

Situated Learning, which was first introduced by Lave and Wenger (39), supports that students learn more effectively when they get involved in realistic environments. In first aid training, using simulated victims helps create these kinds of environments, giving trainees the chance to apply theoretical knowledge in realistic scenarios. Moreover, practicing in a controlled setting allows students to develop their skills and receive quick feedback for improving themselves (40).

Social Constructivism, which was first introduced by Vygotsky (41), emphasizes the importance of interaction in learning processes. During first aid training, practice with simulated victims helps students develop skills, exchange their knowledge, and share their experiences, which improves understanding, practical application, and helps improve their technical skills and how they interact with others.

Game-Based Learning also contributes to the training process through an interactive and engaging structure (42), since role-playing and simulations create a realistic environment for practice without the existence of risks. These activities promote active participation, help students to build confidence and to gain experience in how to handle emergency situations.

## Assessment scales for simulated patients in first aid training

Assessing the performance of simulated patients is an important part of the learning process, since it helps to maintain training quality (38). A common way to evaluate the quality of these interventions is to review the recorded interactions between the students and the simulated victims. This approach has been applied in medical education, during emergency or non-emergency practices, as reviewing interactions between the medical students and the simulated patients the quality of simulation-based and role-playing methods can be ensured. Previous studies have found that evaluations of the performance of simulated victims by the students and their trainers are often correlated. This suggests that students can be improved according to the feedback they receive (43).

To ensure a comprehensive and reliable assessment of simulated patient performance, feedback from students should be gathered right after their performance. This approach gathers different perspectives and experiences, and it helps to identify strengths of the simulation or potential areas for improvement.

A well-known instrument for assessing simulated patient performance is the Standardized Patient Performance Rating Scale-Student Version (SPRS-S), developed by Gonullu et al. (34). This scale consists of nine items and assesses four main factors: patient portrayal, student observation, case recall, and feedback delivery. An important characteristic of SPRS-S is the use of the Angoff method to set the passing threshold for the simulated patient’s performance. Experts have determined this threshold at 24.11/45, which means that the simulated patient must achieve at least this score in order to be considered satisfactory. Moreover, since the scale has a high internal consistency coefficient (Cronbach’s *α* = 0.91), it is suitable for assessing performers quickly when ongoing and effective evaluations are required.

Another useful tool is the Maastricht Assessment of Simulated Patients (MaSP) (44), which focuses on the authenticity of the roles and the quality of feedback provided by the simulated patients. Although this tool provides a more detailed assessment, it has a longer completion time which makes it less practical for quick evaluations. Moreover, the Nijmegen Evaluation of the Simulated Patient (NESP) focuses on assessing interpersonal skills, including how well simulated patients interact with students and provide meaningful feedback (35). This tool, which is commonly used in medical education, is better suited for in-depth evaluations when detailed analysis is required.

Finally, the Objective Structured Assessment of Technical Skills (OSATS) tool is a measurement which was designed to assess how well technical skills are applied in clinical simulations (45). Even though it was originally designed to evaluate students, it can also be adapted to evaluate simulated patients, especially in settings that emphasize technical skills.

All scales mentioned above provide different ways to evaluate simulated patient performance, and each of them focuses on evaluating specific factors. Most of these tools contain 21 to 28 items (35, 44) and they were mainly developed for medical and nursing students. However, adapting and validating these tools for first aid training in school-aged students is essential, since early first aid education is gaining importance year by year (5). As a result developing assessment tools for this task for student populations is considered to be very important.

This study aims to fill this gap by developing and validating a user-friendly, short assessment scale, personalized for use with students, such as the SPRS-S (34). This scale can be applied frequently, particularly when there are many participants and in cases where the evaluations need to be completed quickly after each session. It provides accurate insights into the strengths and weaknesses of the simulation. Consequently, systematic assessment using this method represents a significant area for exploration, contributing to the improvement of the educational process and the quality of simulations.

To achieve this purpose, the following research questions were formulated:

1. Does the Greek version of the SPRS-S scale confirm the unidimensional factor structure of the original scale in a student population?
2. Does the Greek version of the SPRS-S scale demonstrate high internal consistency, making it suitable for use with school-aged students?
3. Can the adapted SPRS-S scale distinguish between different levels of simulated patient performance based on student evaluations?
4. Are the evaluations of the Greek version of the SPRS-S scale influenced by demographic factors, such as the gender or age of the students or the gender of the simulated patient? Moreover, does the scale demonstrate measurement invariance across these demographic subgroups?

The research hypothesis addresses whether the adapted Greek version of the SPRS-S tool can provide reliable and valid measurements for assessing the performance of simulated patients in the context of first aid training. Additionally, it explores whether the factor structure derived from the application of the SPRS-S to the Greek student population will confirm the original theoretical model used in its development.

## Method

### Participants

The study involved a total of 332 students and 3 adult first aid instructors from the National Emergency Aid Center (EKAB). The instructors, two men and one woman, were responsible for teaching students basic first aid skills. Subsequently, through role-playing activities, the instructors acted as simulated patients, allowing the students to apply their acquired knowledge in realistic scenarios.

The sample was selected using purposive sampling, targeting students participating in the first aid educational programs organized by the Center for Environmental Education (KEPEA) of Boeotia. These programs aimed to enhance students’ first aid knowledge and skills, preparing them to manage emergency situations effectively.

Of the total participants, 160 were boys (48%) and 172 were girls (52%), aged between 10 and 16 years. The distribution of students across educational levels was as follows: 14% were in the fifth grade of primary school, 11% in the sixth grade, 11% in the first year of middle school, 14% in the second year of middle school, 10% in the third year of middle school, 13% in the first year of high school, 12% in the second year of high school, and 16% in the third year of high school (see Table 1).

**Table 1:** Frequency (N) and relative frequency (%) of characteristics among student participants in the study.

Participant characteristics	Categories	n	%
Gender	Boy	160	48.19
	Girl	172	51.81
Grade	Fifth grade	45	13.55
	Sixth grade	37	11.14
	First year of middle school	38	11.45
	Second year of middle school	45	13.55
	Third year of middle school	32	9.64
	First year of high school	43	12.95
	Second year of high school	40	12.05
	Third year of high school	52	15.66

### Linguistic adaptation of the scale and item scoring

Before administering the Standardized Patient Performance Rating Scale-Student Version (SPRS-S) to the study participants, the tool underwent conceptual and linguistic adaptation into Greek. This process followed the committee approach, as described in the research of Harkness, Pennell, and Schoua-Glusberg (46). This method was chosen instead of translation-back translation as it ensures better accuracy and consistency in the meaning of the scale’s items.

The process began with two independent translators, both native Greek speakers, translating the scale from English into Greek. Because the SPRS-S was originally developed in Turkey, our team also took into account the Turkish cultural background of the scale. In collaboration with colleagues familiar with Turkish educational and cultural contexts, we verified that any culturally specific nuances were accurately captured. Each translator created a separate version, which the research team then reviewed and compared. Following a detailed discussion, the translators and the research team agreed on a final version that aligned closely with both the meaning and the cultural context of the original scale.

Two bilingual experts with experience in psychometric assessment and measurement then reviewed this initial version. Both had international experience from their professional work, and they did not have access to the original scale to ensure unbiased evaluation. Their feedback led to necessary revisions.

In the final stage, the research team worked with the translators to finalize the Greek version of the tool, ensuring it was suitable for the study. This final version was then administered to the participants, and the results of its use were analyzed in the context of the current research (see Appendix).

Participants’ responses were recorded on a four-point Likert-type scale, enhanced with graded facial expressions to represent levels of agreement: (a) Strongly Disagree, (b) Disagree, (c) Agree, (d) Strongly Agree. This approach leveraged the expressiveness of icons to simplify responses and ensure accurate reflection of participants’ perceptions.

### Procedure

The study began with the training of student groups by one of the three instructors from the National Emergency Aid Center (EKAB) during the 2023–24 and 2024–25 school years. The training sessions were held at the facilities of the Center for Environmental Education (KEPEA) of Boeotia and lasted 2 h each. Each session followed a structured format that included a brief introduction to first aid principles, demonstration and enactment of selected emergency scenarios, and short guided reflection. The sessions focused on emergency situations such as cardiopulmonary resuscitation (CPR), managing respiratory distress (asphyxia, drowning, or asthma attacks), providing assistance during epileptic seizures, addressing fainting episodes, and performing the Heimlich maneuver to clear airway obstructions. Due to time constraints and the educational design of the program, not all emergency situations were enacted by every student; instead, students participated in and observed a subset of scenarios representative of common first aid situations.

The study was conducted in accordance with ethical standards for research involving human participants. Ethical approval was granted by the Ethics and Bioethics Committee of the Center for Environmental Education (KEPEA) of Boeotia (F.4.1./62/16-3-2024). Written informed consent was obtained from the legal guardians of all participating students prior to data collection.

In addition, the training process involved the instructors acting as simulated patients, replicating the aforementioned emergency scenarios. This provided students with the opportunity to practice in a safe yet realistic environment, applying the practical skills they had learned. This role-playing approach enhanced the learning experience and facilitated the practical application of knowledge.

Data collection took place immediately after the completion of each scenario. Students evaluated the instructor’s performance as a simulated patient based on the specific scenario they had participated in or observed, using the SPRS-S scale, which had been adapted into Greek for this study. For the purposes of the subsequent psychometric analyses, the total student sample was randomly divided into two independent subsamples.

### Data analysis

In the first group (*N* = 170), Exploratory Factor Analysis (EFA) was performed to identify the underlying factor structure (47). In the second group (*N* = 162), Confirmatory Factor Analysis (CFA) was conducted to validate the factor structure suggested by the original developers of the scale. Initially, the suitability of the sample for factor analysis was assessed using the Kaiser-Meyer-Olkin (KMO) measure, while Bartlett’s test of sphericity was used to evaluate the adequacy of inter-item correlations. These tests ensured that the data met the necessary conditions for factor analysis. The factor estimation method used in CFA was Maximum Likelihood (ML), which assumes multivariate normality. However, given the relatively large sample size (*N* = 332), Maximum Likelihood (ML) estimation remains appropriate, as minor violations of normality do not substantially impact factor loading accuracy (48).

Next, the readability of the Greek version of the scale was evaluated using the Flesch–Kincaid and Gunning Fog indices to determine whether the linguistic content was appropriately tailored to the target group.

To assess the reliability of the adapted scale, Cronbach’s Alpha (*α*) and McDonald’s Omega (*ω*) were calculated. Moreover, a clustering technique was used, based on a nature-inspired metaheuristic optimization algorithm known as the Flying Foxes Optimization (49, 50) on the total sample. This technique classified performance into low, medium, and high levels. This methodology was used because metaheuristic algorithms perform better than traditional analytical methods in clustering problems. Their advantage comes from their ability to explore solution spaces more effectively, especially when the data are complex and non-linear, allowing them to avoid local minima and examine the solution space more broadly (51–55).

Finally, the gender and the age-related differences among student participants were examined, along with performance differences based on the instructor’s gender. Data analysis was conducted using Jamovi 2.3.17 software. A multi-group CFA was conducted to test the measurement invariance of the SPRS-S across gender. The lavaan package in R (version 4.3.2) was used for this analysis (56). A multi-group confirmatory factor analysis (multi-group CFA) is a statistical technique used to test whether a measurement model operates equivalently across different groups (e.g., gender, age, cultural backgrounds) (57).

## Results

Table 2 presents descriptive statistics for the nine items of the Greek SPRS-S scale, revealing an overall positive evaluation by the participants (*N* = 332).

**Table 2:** Descriptive measures of responses to the items of the Greek version of the SPRS-S scale.

Item	Mean	SD	Std. error mean	95% CI mean		Min	Max	Item-rest correlation
				LL	UL			
Q1	3.36	0.62	0.03	3.30	3.43	1	4	0.65
Q2	3.42	0.60	0.03	3.36	3.49	1	4	0.69
Q3	3.30	0.63	0.03	3.23	3.37	1	4	0.67
Q4	3.19	0.59	0.03	3.13	3.25	1	4	0.6
Q5	3.39	0.62	0.03	3.32	3.46	1	4	0.65
Q6	3.33	0.60	0.03	3.26	3.39	1	4	0.65
Q7	3.24	0.62	0.03	3.18	3.31	1	4	0.7
Q8	3.37	0.62	0.03	3.30	3.44	1	4	0.62
Q9	3.49	0.59	0.03	3.42	3.55	1	4	0.6

After the sample was randomly divided into two groups, the first group (*N* = 170) was evaluated to determine its suitability for Exploratory Factor Analysis (EFA). This was confirmed using the Kaiser-Meyer-Olkin (KMO) measure, which indicated excellent overall sampling adequacy (KMO = 0.93). Furthermore, individual KMO values ranged from 0.92 to 0.94, highlighting the adequacy of correlations between variables. Bartlett’s test of sphericity was also statistically significant (*χ*^2^(36) = 1249.81, *p* < 0.001), indicating that inter-item correlations were strong enough to justify the application of EFA.

**Figure 1:**
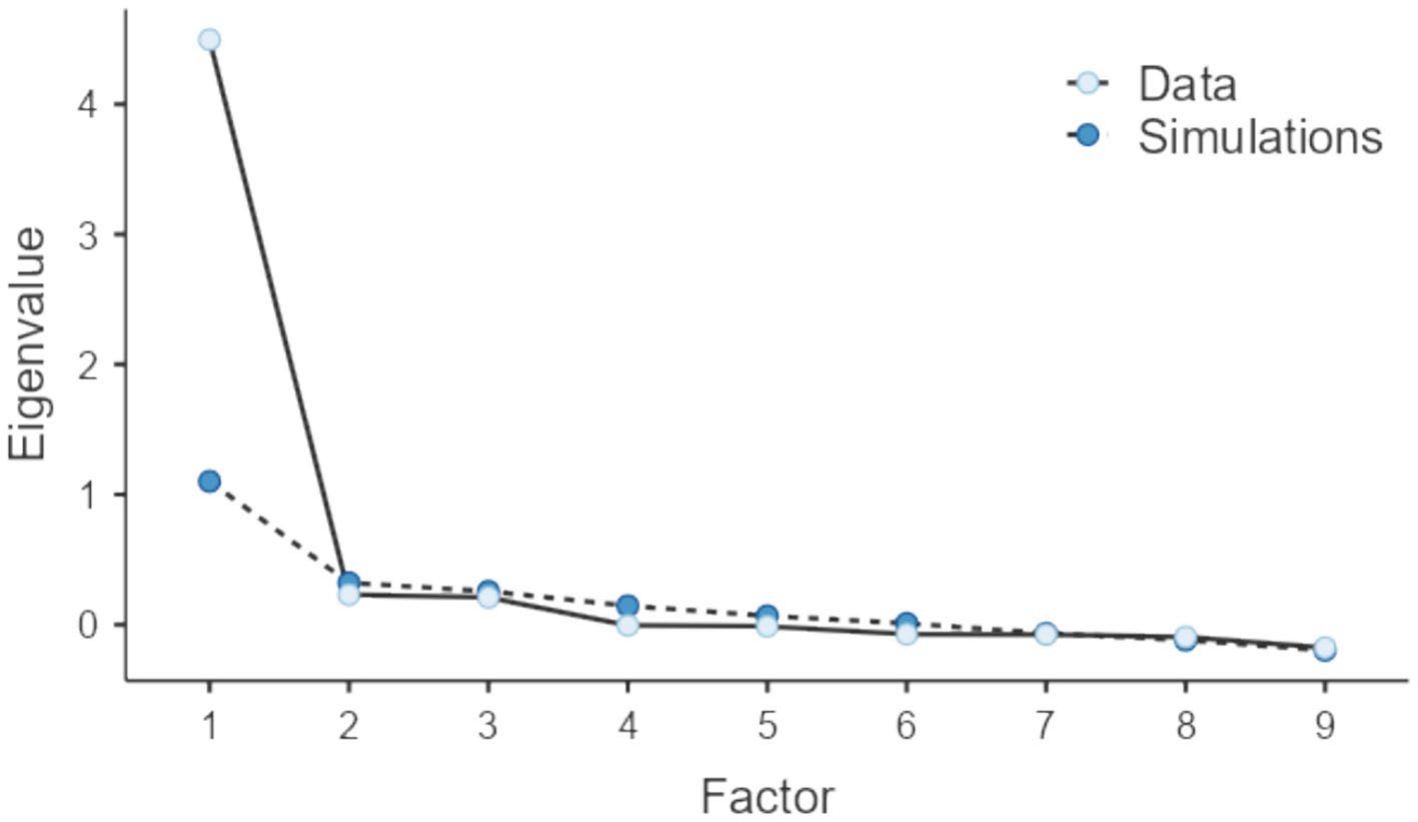
Selection of number of factors based on Horn’s parallel analysis criterion.

The results of the EFA supported the selection of a single factor, based on the outcomes of parallel analysis (64). This factor accounted for 49.95% of the covariance among the analyzed variables (see Figure 1). This value was deemed satisfactory, as per a general guideline suggesting that values near 50% are adequate for explaining covariance and should be accepted (58).

The specific variances (𝜎^2^) for the nine items of the factor ranged from 0.42 to 0.64, while factor loadings varied between 0.60 and 0.76, with an eigenvalue of 4.50 (see Table 3).

**Table 3:** Factor matrix.

Item	Loadings	σ^2^
Q7	0.76	0.42
Q2	0.74	0.45
Q5	0.74	0.45
Q6	0.74	0.45
Q8	0.71	0.49
Q3	0.70	0.50
Q1	0.70	0.51
Q9	0.65	0.58
Q4	0.60	0.64

Confirmatory Factor Analysis (CFA) conducted on the second sample (*N* = 162), confirmed the unidimensional structure of the SPRS-S scale. All factor loadings were statistically significant (*p* < 0.001) and ranged from 0.34 to 0.48, indicating a strong relationship between the items and the underlying factor (see Table 4).

**Table 4:** Factor loadings from confirmatory factor analysis.

Indicator	Estimate	SE	Z	*p*
Q1	0.45	0.05	9.59	< 0.001
Q2	0.42	0.04	10.07	< 0.001
Q3	0.48	0.05	10.72	< 0.001
Q4	0.41	0.04	9.49	< 0.001
Q5	0.38	0.04	8.66	< 0.001
Q6	0.37	0.04	8.51	< 0.001
Q7	0.48	0.05	10.45	< 0.001
Q8	0.35	0.05	7.67	< 0.001
Q9	0.34	0.04	8.65	< 0.001

The model fit indices showed good alignment with the data, with values as follows: CFI = 0.96, TLI = 0.95, SRMR = 0.04, and RMSEA = 0.07 (90% CI: 0.04–0.10). The chi-square to degrees of freedom ratio was 1.79 (*χ*^2^ = 48.37, df = 27), which falls within acceptable limits (*χ*^2^/df < 3), further supporting the model’s fit (59, 60). As commonly observed, standardized CFA loadings were lower than EFA loadings, due to the more restrictive assumptions of confirmatory modeling and the separation of common and error variance. The reliability of the Greek SPRS-S scale was tested on the full sample (*N* = 332). Cronbach’s alpha was 0.89 and McDonald’s omega also 0.89.

These findings indicate that the Greek version of the SPRS-S scale demonstrates strong factorial validity and fits well with the sample data. The readability analysis of the final Greek version showed that the scale is easy to understand for students aged 10 and above as the Flesch–Kincaid readability score was 71.55, and the Gunning Fog index was 61.55 (61).

With a mean score of 3.34 (SD = 0.45), the results reflect a generally positive assessment of the participants. For this reason, a multivariate clustering was performed on the total sample (*N* = 332) using the metaheuristic Flying Foxes Optimization algorithm (49) identified three performance levels. The Davies–Bouldin Index (DBI) score for the clustering was 0.52. The low level corresponded to mean scores up to 3 and included 89 individuals. The medium level ranged from mean scores above 3 and up to 3.67 and included 166 individuals. The high level comprised mean scores above 3.67, with 77 individuals categorized in this group.

Finally, independent-sample t-tests revealed no statistically significant differences in SPRS-S mean scores between boys and girls (*t*(330) = 0.63, *p* = 0.529). Similarly, an analysis of variance (ANOVA) investigating differences in mean scores across educational levels, as an indicator of age, showed no statistically significant differences *F*(7,324) = 1.08, *p* = 0.373. An additional independent-sample t-test evaluating differences based on the gender of the simulated patient also showed no significant results (t(330) = −0.11, *p* = 0.909). These findings strengthen the validity of the SPRS-S scale, confirming that it is unaffected by factors such as the students’ age, gender, or the gender of the simulated patient.

When Table 5 is examined, it is observed that the parameter values obtained for the structural invariance stage fall within the appropriate cutoff ranges (CFI > 0.97, TLI > 0.96, SRMR < 0.04, and RMSEA < 0.10). The factor structures and patterns were found to be similar across gender, and it was determined that the SPRS-S meets structural invariance for both girls and boys.

**Table 5:** Measurement invariance results from confirmatory factor analysis.

Stages of measurement invariance	𝜒^2^	df	CFI	TLI	SRMR	RMSEA (%90 C. I.)	*∆*CFI
Structural invariance	98.987	54	0.969	0.958	0.037	0.066(0.042–0.088)	-
Metric invariance	99.785	62	0.970	0.965	0.049	0.061(0.037–0.082)	0.001
Scalar invariance	110.059	70	0.968	0.967	0.052	0.059(0.036–0.079)	0.002
Strict invariance	134.282	79	0.955	0.959	0.058	0.066(0.045–0.083)	0.013

In the metric invariance stage, the analysis results indicated that the parameter values did not deviate from the acceptable range (CFI > 0.97, TLI > 0.97, SRMR < 0.05, RMSEA < 0.10, and ∆CFI < 0.01). Additionally, in the ANOVA comparisons, the AIC and BIC values were found to be lower for metric invariance. Based on these findings, it can be stated that the factor loadings of SPRS-S do not vary by gender and that metric invariance is achieved. Following the confirmation of metric invariance, scalar invariance was analyzed.

For the examination of scalar invariance, the parameters obtained from the analysis were compared with the cutoff values (CFI > 0.968, TLI > 0.967, SRMR < 0.052, RMSEA < 0.10, and ∆CFI < 0.01), and it was observed that SPRS-S falls within the acceptable limits for scalar invariance across gender. ANOVA comparisons also showed that AIC and BIC values were lower for scalar invariance. Based on these findings, it can be stated that the regression intercepts of SPRS-S do not differ across gender subgroups. Following the confirmation of scalar invariance, the strict invariance stage was tested.

The analysis of SPRS-S in terms of strict invariance indicated that although the CFI, TLI, SRMR, and RMSEA values remained within the acceptable thresholds, the ∆CFI value (∆CFI > 0.01) was found to be outside the acceptable limit. Similarly, ANOVA comparisons showed that the BIC value was lower for strict invariance. These findings suggest that SPRS-S does not achieve strict invariance.

Overall, since scalar invariance has been established, it can be stated that comparisons across the relevant subgroups can be made without introducing systematic error. If scalar invariance is met, it implies that the test items do not show bias for different subgroups. Conversely, this information can be interpreted as follows: a test that achieves scalar invariance contains unbiased items for different subgroups (62).

## Discussion

This study aimed to adapt and psychometrically validate the SPRS-S scale in Greek to assess its suitability for use with a student population in the context of first aid training. The findings support the unidimensional factor structure of the scale, which proved appropriate for implementation in a student population and within the Greek context. This result directly addresses the first research question, indicating that the construct of simulated patient performance is captured consistently as a single latent dimension when evaluated by school-aged students.

The reliability results of the scale, as indicated by high Cronbach’s alpha and McDonald’s omega values, confirm the internal consistency of its items and reveals that the scale measures a single factor effectively. This high reliability complies with other tools for assessing simulated patients and with the original scale (34, 44), thereby validating the Greek version as a dependable tool for educational assessment. In practical terms, this suggests that students are able to provide stable and coherent evaluations of simulated patient performance, despite their age and limited prior experience with formal assessment procedures. Additionally, linguistic adaptation ensures that students fully understand the questions, which is essential for the effective use of assessment tools in school settings (5, 61).

Through the adapted SPRS-S, one can observe three different performance levels in the simulated patient role, classifying them as low (mean scores of 3 or lower), medium (between 3 and 3.67), and high (above 3.67). This finding responds to the third research question and indicates that the scale is sensitive enough to differentiate between varying degrees of performance. This differentiation is what makes the scale useful, since it allows instructors to assess specific strengths while also identifying areas that may need improvement. Such differentiation is particularly important in first aid education, where the quality of role enactment and feedback delivery can substantially influence students’ learning and confidence (1). Finally, it contributes to the overall effectiveness of first aid training, ensuring that participants receive constructive feedback to optimize their performance.

The results showed that evaluations were not affected by students’ gender or age, nor by the gender of the simulated patient. The results of the multi-group CFA analysis indicate that the SPRS-S demonstrates structural, metric, and scalar invariance across gender, suggesting that the factor structure, factor loadings, and regression intercepts remain stable for both male and female participants. This finding addresses the fourth research question and supports the interpretation that the scale functions equivalently across key demographic subgroups. Since scalar invariance was achieved, comparisons of latent mean scores between gender groups can be made without bias, ensuring that any observed differences reflect true variations rather than measurement artifacts. This reveals the scale’s strengths and demonstrates that it can be applied to different groups. This is very important since ensuring fairness in assessment methods is essential to maintain their validity and ensure that they continue to be effective when being applied to different groups (63). As a result, this scale is a more reliable option since it remains neutral, unlike other scales that might be affected by such variables.

Despite the results which suggest that the Greek version of the SPRS-S is a useful tool for assessing simulated patients and supporting learning in first aid education, our research also has limitations. A possible limitation of our research is that it includes only students in schools. As a result, the results may not be the same when the scale is applied in older populations or other educational settings such as higher education. In addition, the use of simulated patients portrayed by instructors, while pedagogically appropriate, may differ from professional standardized patient programs, which could influence performance ratings. Future research should test the scale on different groups (like medical or nursing students) or in more advanced scenarios, as well as examine its predictive validity in relation to long-term skill retention through delayed post-training assessments.

## Conclusion

The present study provides empirical evidence supporting the use of the Greek version of the SPRS-S scale for evaluating simulated patient performance in school-based first aid training. The scale demonstrates a clear unidimensional structure, high reliability, and the capacity to distinguish between different levels of performance, indicating that it functions consistently and meaningfully within a student population. These characteristics make it suitable for evaluating the enactment and feedback quality of simulated patients in educational contexts.

Finally, the scale can help create evaluation protocols for first aid training programs to which performance assessment is useful. Moreover, the use of the scale allows students to receive clear and useful feedback and encourages collaboration between students and instructors. As a result, it can promote a learning environment that focuses on safety and quick response in first aid education.

## Data Availability

The dataset supporting the conclusions of this article is publicly available in the Harvard Dataverse repository at https://doi.org/10.7910/DVN/JJ0C6Z. The data are fully anonymized.

## Appendix

Items of the Greek Version of the SPRS-S Scale:

1. Αυτός που παίζει τον ασθενή κάνει τον ρόλο του σαν να είναι αληθινός.
  *(The simulated patient performs their role as if it were real).*
2. Είναι εύκολο να τον καταλάβω τον ρόλο αυτού που παίζει τον ασθενή.
  *(It is easy to understand the role of the simulated patient).*
3. Οι απαντήσεις αυτού που παίζει τον ασθενή στις ερωτήσεις μου είναι σωστές.
  *(The simulated patient’s answers to my questions are accurate).*
4. Τα σχόλια αυτού που παίζει τον ασθενή με βοηθούν να καταλάβω πώς τα πήγα.
  *(The simulated patient’s feedback helps me understand how I performed).*
5. Αυτός που παίζει τον ασθενή μου δίνει παραδείγματα από το παιχνίδι ρόλων.
  *(The simulated patient provides examples from the role-play).*
6. Αυτός που παίζει τον ασθενή με βοηθάει να κάνω ερωτήσεις.
  *(The simulated patient helps me ask questions).*
7. Αυτός που παίζει τον ασθενή λέει πώς ένιωσε στην άσκηση.
  *(The simulated patient explains how they felt during the exercise).*
8. Αυτός που παίζει τον ασθενή μου δίνει ιδέες για να τα πάω καλύτερα.
  *(The simulated patient gives me suggestions for improvement).*
9. Αυτός που παίζει τον ασθενή μου μιλάει ευγενικά.
  *(The simulated patient speaks to me kindly).*
